# Characterization of Polyhydroxybutyrate, PHB, Synthesized by Newly Isolated Haloarchaea *Halolamina* spp.

**DOI:** 10.3390/molecules27217366

**Published:** 2022-10-29

**Authors:** Nashwa Hagagy, Amna A. Saddiq, Hend M. Tag, Samy Selim, Hamada AbdElgawad, Rosa María Martínez-Espinosa

**Affiliations:** 1 Department of Biology, College of Science and Arts at Khulis, University of Jeddah, Jeddah 21959, Saudi Arabia; 2 Department of Clinical Laboratory Sciences, College of Applied Medical Sciences, Jouf University, Sakaka 72388, Saudi Arabia; 3 Botany and Microbiology Department, Faculty of Science, Beni–Suef University, Beni–Suef 62521, Egypt; 4 Biochemistry and Molecular Biology Division, Department of Agrochemistry and Biochemistry, Faculty of Science, University of Alicante, Carretera San Vicente del Raspeig s/n-03690 San Vicente del Raspeig, E-03690 Alicante, Spain; 5 Multidisciplinary Institute for Environmental Studies “Ramón Margalef”, University of Alicante, Ap. 99, E-03080 Alicante, Spain

**Keywords:** haloarchaea, polyhydroxybutyrate (PHB), polyhydroxyalkanoate (PHA), *Halolamina*, FTIR, HPLC

## Abstract

This work aims to characterize the haloarchaeal diversity of unexplored environmental salty samples from a hypersaline environment on the southern coast of Jeddah, Saudi Arabia, looking for new isolates able to produce polyhydroxyalkanoates (PHAs). Thus, the list of PHA producers has been extended by describing two species of *Halolamina*; *Halolamina sediminis* sp. strain NRS_35 and unclassified *Halolamina* sp. strain NRS_38. The growth and PHA-production were investigated in the presence of different carbon sources, (glucose, sucrose, starch, carboxymethyl cellulose (CMC), and glycerol), pH values, (5–9), temperature ranges (4–65 °C), and NaCl concentrations (100–350 g L^−1^). Fourier-transform infra-red analysis (FT-IR) and Liquid chromatography–mass spectrometry (LC-MS) were used for qualitative identification of the biopolymer. The highest yield of PHB was 33.4% and 27.29% by NRS_35 and NRS_38, respectively, using starch as a carbon source at 37 °C, pH 7, and 25% NaCl (w/v). The FT-IR pattern indicated sharp peaks formed around 1628.98 and 1629.28 cm^−1^, which confirmed the presence of the carbonyl group (C=O) on amides and related to proteins, which is typical of PHB. LC-MS/MS analysis displayed peaks at retention times of 5.2, 7.3, and 8.1. This peak range indicates the occurrence of PHB and its synthetic products: Acetoacetyl-CoA and PHB synthase (PhaC). In summary, the two newly isolated *Halolamina* species showed a high capacity to produce PHB using different sources of carbon. Further research using other low-cost feedstocks is needed to improve both the quality and quantity of PHB production. With these results, the use of haloarchaea as cell factories to produce PHAs is reinforced, and light is shed on the global concern about replacing plastics with biodegradable polymers.

## 1. Introduction

The concept “plastic” usually refers to numerous organic synthetic or processed materials mainly obtained from petroleum that are not biodegradable. They are mostly thermoplastic or thermosetting polymers of high molecular weight. The extensive use of plastics manufactured from petroleum (which are non-biodegradable and recalcitrant) is a major threat to the world due to their negative impact on the environment as well as on living beings. For this reason, global contamination by plastics has become the most important problem in the world, which must be radically solved [1].

One of the solutions to the problem of petroleum plastics is to replace them with environmentally friendly, renewable, economically profitable, and biodegradable plastics. Bioplastics are biopolymers made up of a variety of compounds, with most of them being renewable, such as cellulose, starch, or bioethanol [2]. They are produced and accumulated by several microorganisms, such as bacteria, cyanobacteria, and archaea, as intracellular granules and serve as carbon storage for energy [3].

Among them, polyhydroxyalkanoates (PHA) are polyesters synthesized by several microorganisms. PHAs occur naturally and accumulate intracellularly in microorganisms as storage granules with physicochemical characteristics similar to petrochemical polymers [4]. The most common PHA is the PHB (polyhydroxybutyrate), which is highly marketed due to its outstanding optical characteristics and great UV resistance [5]. Thus, several PHA types have been highlighted for being biostable, environmentally safe, and ecologically feasible with a spellbinding performance of nontoxicity, moldability, crystallinity, thermoplasticity, and composability [6]. These unique and distinctive polymers have covered many fields as they have been applied in the medical field (as medical materials and drug carriers) as well as in the agriculture and packaging industries.

Extremophilic microorganisms, particularly haloarchaea, show all the optimum features to be a good candidate to produce PHA on a large scale: Microbial growth can be achieved by using waste carbon sources and there is no need for medium sterilization, minimal risk of contamination by other microorganisms, use of brines or seawater for medium preparation, recovery of the salts used in the medium, and a simple cell lysis process with water to harvest the polymer granules [7,8]. Consequently, as bioplastic producers, haloarchaea have advantages over other bacterial strains, hence minimizing the production cost of PHA [9,10]. Consequently, finding new PHA producers within haloarchaea is crucial for cost-effective polymer manufacturing. Thus, this subject has attracted attention during the last decade, aiming at the isolation of new strains able to synthesize PHA.

For example, some haloarchaeal strains belonging to genera such as *Haloquadratum, Halobiforma, Halorhabdus, Halobacterinm, Halococcus, Halopiger, Haloferax, Natrinema,* and *Natronorubrum* are PHA producers under certain nutritional conditions and do not require sterile conditions (due to high salt concentration) for growth, which makes them interesting species to be used as cell factories to produce PHAs at a large scale [11,12]. Polyhydroxybuttyrate (PHB) and Polyhydroxybutyrate-valerate (PHBV) (PHB-copolymer that shows lower crystallinity degree and more flexibility than PHB) can be biosynthesized via three species of the *Haloferax* genus (*Hfx. mediterranei, Hfx. volcanii,* and *Hfx. gibbonsii*) and three species of the *Haloarcula* genus (*Har. hispanica, Har. vallismortis, and Har. japonica*) [13,14]. Furthermroe, *Halopiger aswanensi* was found to accumulate PHB for approximately 53% of its cell dry weight by using n-butyric acid and sodium acetate as sources of carbon [15,16]. Two species of *Haloquadratum walsbyi* and other species belonging to *Halostagnicola, Haloterrigena, Halobiforma, Haloarcula, Halobacterium, Halocococcus, Halorubrum, Natrinema,* and haloalkaliphiles that include *Natronobacterium* and *Natronococcus* have also shown the capacity to produce PHA [17,18,19,20]. The most abundant species in Antarctica’s Deep Lake, *Halorubrum lacusprofundi*, was recently reported to be a PHA-like granule-producer at low temperatures [21].

Considering the previous promising works using haloarchaea as cell factories to produce these bioplastics, the aim of this work is to explore the haloarchaeal diversity of environmental salty samples taken in the Saudi Arabia Kingdom with special attention paid to new isolates able to produce PHA. Optimization of microbial growth and PHA production using two newly isolated *Halolamina* species has been carried out. This work sheds light on PHA production by haloarchaea and contributes to the knowledge about their use as cellular factories to produce those polymers under circular economy approaches, thus contributing to environmentally friendly processes that may replace the chemical synthesis of plastics.

## 2. Results

### 2.1. Isolation, Screening, and Identification of Haloarchaeal Strains Showing PHA-Producing Capacities

In this study, low-cost carbon sources were used to screen PHA-producing haloarchaea isolated from a Solar Saltern in Jeddah, Saudi Arabia. To identify the presence of PHA granules, traditional staining methods (Sudan Black B) were used. Among twelve isolates, two potential strains, NRS 35 and NRS 38, demonstrated the presence of black granules when stained with Sudan Black B, thus corroborating their capability as PHA producers [7]. More phenotypic characteristics were explored and are shown in Table 1.

**Figure 1:**
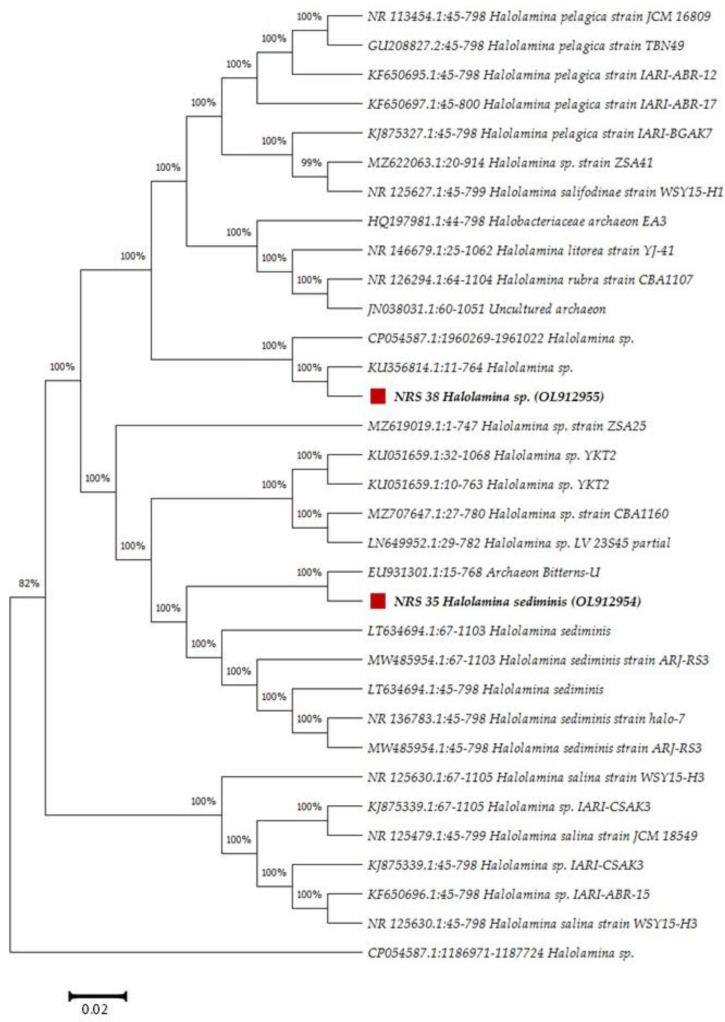
Maximum likelihood phylogenetic tree based on 16S rRNA gene sequences showing the relationship between the potential PHB-producers NRS_35 and NRS_38 and closely related species from the GenBank database. The scale bar indicates 0.02 substitutions per nucleotide position.

16S rRNA gene sequencing revealed that the selected strains are members of *Halolamina sediminis* showing 96.42% and 96.91% sequence similarity, respectively. The obtained 16S rRNA gene data have been deposited in the NCBI and GenBank nucleotide sequence databases under the accession number OL912954 for *Halolamina sediminis* strain NRS_35 and OL912955 for *Halolamina* sp. strain NRS_38 (Figure 1). *Halolamina* is reported to be a halophilic organism that is widely distributed in marine environments [22].

### 2.2. Optimization of Growth Conditions and PHA Production

**Figure 2:**
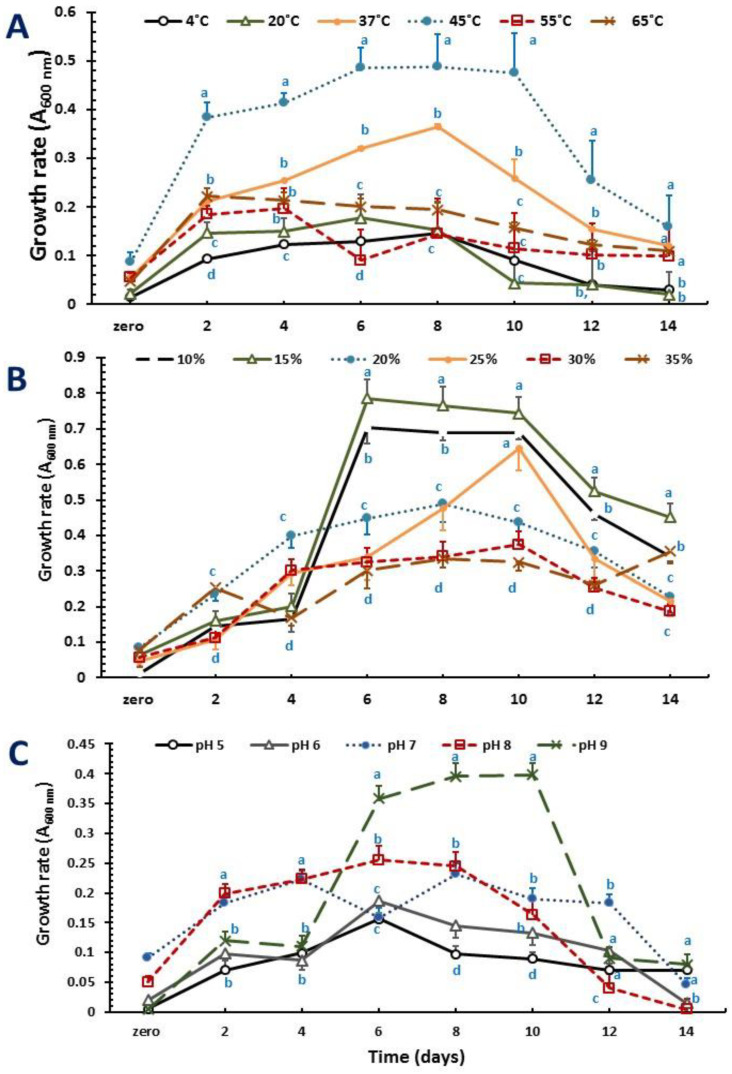
Effect of physical factors on the growth of PHA-producer *Halolamina sediminis* strain NRS_35. Cells were grown in a PHA-production medium supplemented with glycerol (10 g L^−1^) as a carbon source; (**A**) temperature, (**B**) NaCl concentrations, (**C**) pH. For each time, different superscript letters indicate significant differences at the *p* < 0.05 level (one-way ANOVA and Duncan post hoc test) comparing physical factors at different levels.

**Figure 3:**
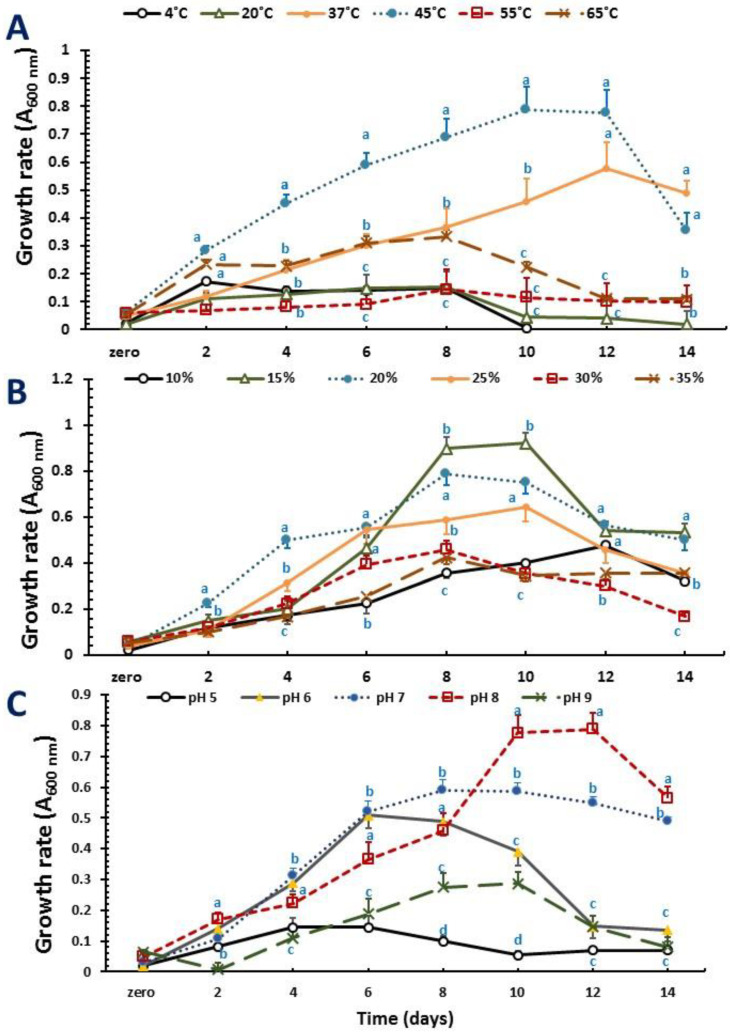
Effect of physical factors on the growth of PHA-producer unclassified *Halolamina* sp. strain NRS_38 grown. Cells were grown in a PHA-production medium supplemented with glycerol (10 g L^−1^) as a carbon source; (**A**) temperature, (**B**) NaCl concentrations, and (**C**) pH. For each time, different superscript letters indicate significant differences at the *p* < 0.05 level (one-way ANOVA and Duncan post hoc test) comparing physical factors at different levels.

**Figure 4:**
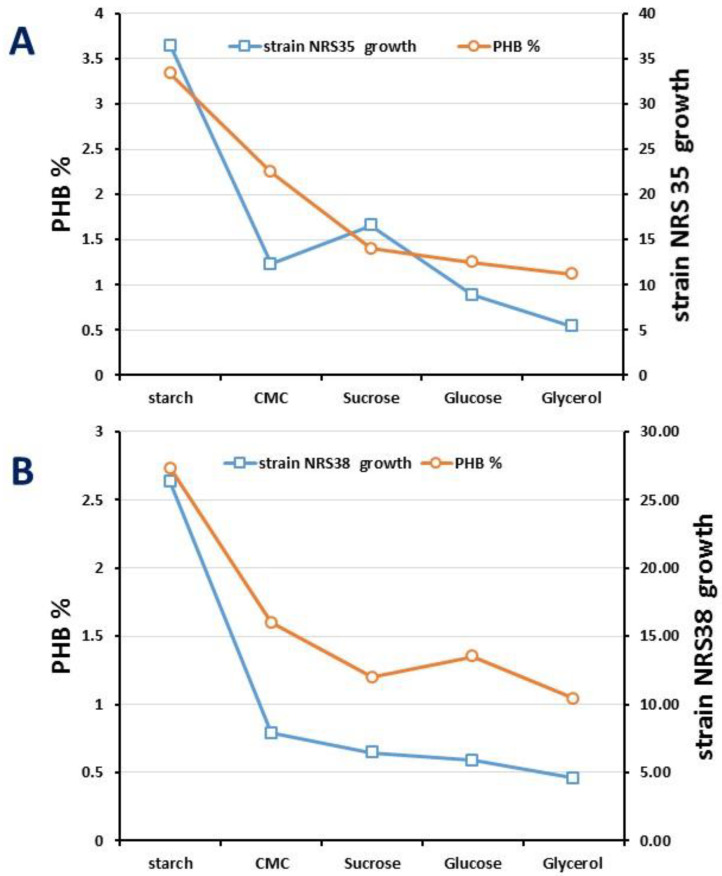
Growth and PHB yield by two new isolates *Halolamina* (**A**) NRS_35 and (**B**) NRS_38 under similar conditions with different carbon sources.

The growth patterns of the two novel strains (NRS_35 and NRS_38) producing PHA granules were monitored on a PHA-production medium including glycerol as a carbon source at various temperatures, salinity, and pH values (Figure 2 and Figure 3). The strains grew at temperatures ranging from 4 to 65 °C. The best temperature for growth was 45 °C for both strains (Figure 2A and Figure 3A). Regarding the concentration of NaCl used during cultivation, the results are displayed in Figure 4: Both strains grew in the presence of NaCl at concentrations ranging from 10% to 35% (w/v) with optimum growth at 15% (Figure 2B and Figure 3B). Related to pH, strains NRS_35 and NRS_38 grew at a pH range of 5–9, with an optimum of 9 (Figure 2C and Figure 3C).

Considering that the availability of carbon and P and the ratio C/N are crucial to optimizing the synthesis of PHA, the effect of various carbon sources on cell growth and PHA biosynthesis in both strains was studied. The results showed that both isolated strains could synthesize PHA from a series of carbon sources including starch, CMC, glucose, glycerol, and sucrose (Figure 4). For *Halolamina sediminis* NRS_35, starch, CMC, sucrose, glucose, and glycerol yielded PHA contents of 33.4%, 22.5%, 14.0%, 12.5, and 11.2, respectively. Regarding the production of PHA by NRS_38, the current results revealed that PHA contents yielded for starch, CMC, glucose, glycerol, and sucrose were equal to 27.29%, 16%, 12%, 13.5%, and 10.4%, respectively. Thus, starch was the most favorable substrate for PHA accumulation by both strains NRS_35 and NRS_38, reaching a titter of 3.6457 and 2.36 g/L, respectively.

The yield of PHA obtained by two different species of *Halolamina* strains, NRS_35 and NRS_38, under similar conditions with different carbon sources is summarized in Table 2. The results revealed that the maximum production of PHA in the presence of starch by NRS_35 is equal to 41.67 ± 2.10 mg/L, which is a higher concentration than the produced by NRS_38 under the same growth conditions. Thus, starch was the most suitable carbon source for biomass production for the tested strains.

### 2.3. FTIR Analysis

**Figure 5:**
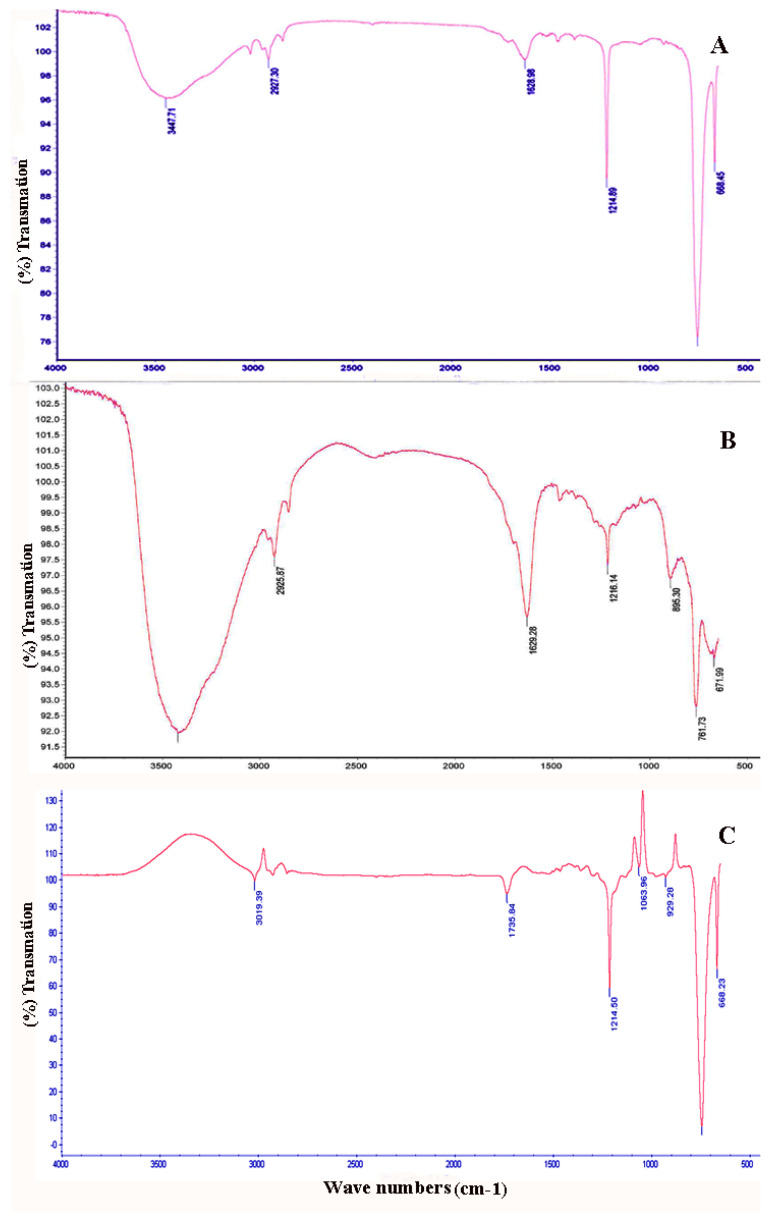
Recorded FT-IR patterns for extracted PHB/PHA (**A**) unclassified *Halolamina* sp. strain NRS_35, (**B**) *Halolamina sediminis* sp. strain NRS_38, and (**C**) standard PHB.

FTIR scan analysis has been used to illustrate the chemical functional groups within biomass yield. The current FTIR pattern (at 4000–400 cm^−1^) for both *Halolamina sediminis* strain NRS_35 and *Halolamina* sp. strain NRS_38 is illustrated in Figure 5A,B, respectively. The wide range of absorption peaks detected around 3447.71 and 3442.84 cm^−1^ indicated the presence of intensive –NH or –OH group bonds in a protein. The weak absorption at 2958.39 cm^−1^ in the unclassified *Halolamina* sp. strain NRS_38 (Figure 5A) is related to the C–H stretching of the –CH_3_ group in fatty acids, while the peaks around 2927.30 and 2925.87 cm^−1^ were generated by the symmetric stretch of C-H bond in CH_2_ groups. The sharp peaks that formed around 1628.98 and 1629.28 cm^−1^ could be related to the carbonyl (-C=O) group on amides and related to proteins or may be associated with the –CONH– group in amino sugars and proteins.

### 2.4. LC–MS/MS Analysis

**Figure 6:**
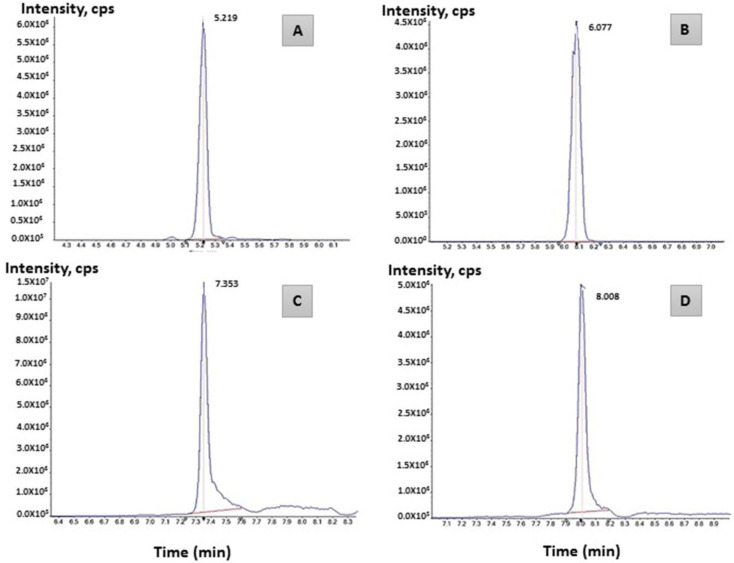
LC-MS spectra of Polyhydroxybutyrate standard diluted in Chloroform. Retention times (in minutes): (**A**) acetyl-CoA [RT: 5.219], (**B**) PhaB [RT: 6.077], (**C**) PhaC [RT: 7.353], (**D**) PHB [RT: 8.008].

**Figure 7:**
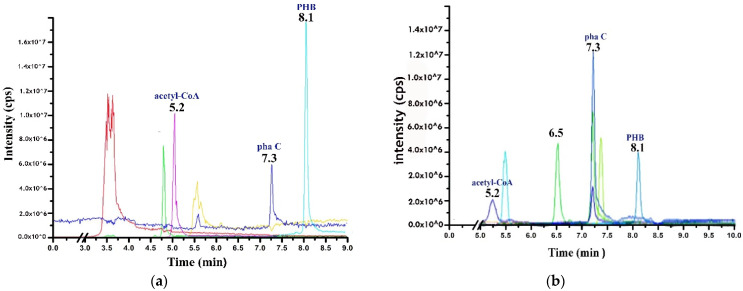
Spectrum from LC-MS/MS analysis of extracted PHB granules from (**a**) NRS_35 and (**b**) NRS_38.

As shown in Figure 6, extracted polymers produced by the NRS_35 strain displayed peaks at different retention times (5.2, 7.3, and 8.1). This peak range indicates the occurrence of PHB and biomolecules involved in its synthesis such as acetoacetyl-CoA (precursor) and the enzyme PHB synthase (PhaC). The detection of PHB in granules produced by NRS_35 and NRS_38 strains using LC-MS/MS is shown in Figure 7a,b, respectively. Regarding the PHB isolated from the NRS_38 strain, peaks are detected at 8.1 min (Figure 7), and at approximately 5.2 and 7.3 min, the last two peaks corresponding to acetyl-CoA and PhaC, respectively, are also observed. Other peaks were observed, which may contain the digested cell debris and other molecules that do not bind to the Accucore C18.

## 3. Discussion

Biodegradable polymers are an alternative to address the challenges related to plastic waste [23]. Among these biopolymers, polyhydroxyalkanoates (PHAs) are excellent candidates to be explored, mainly PHB and PHBV, due to their physicochemical characteristics [5]. Some archaea that are extremely halophilic can synthesize and accumulate PHA as granules within their cells [24]. Within this context, several previous works reported that several haloarchaeal genera can use different types of carbohydrates as sources of carbon and energy for PHB production [5]. This production can be optimized by modulating the C/N ratio and phosphate availability or/and by obtaining mutants overexpressing enzymes involved in PHA synthesis [5].

In this study, the goal was to screen haloarchaea capable of PHA-production from environmental samples taken from a Solar Saltern located in Jeddah, Saudi Arabia. To conduct this research, low-cost carbon sources have been used as nutrients in order to optimize an environmentally friendly method that could be further related to circular economy approaches. Out of ten haloarchaeal isolates, two strains, NRS_35 and NRS_38, exhibited the presence of black granules when stained with Sudan Black B, indicating their capability of PHA biosynthesis [25].

The growth profile of both strains was monitored on a PHA-production medium including glucose, among other molecules, as a carbon source and modifying several parameters such as the temperature, pH values, and salt concentration. The results obtained revealed that the optimum temperature for NRS_35 and NRS_38 growth was 45◦C. Both strains grew in the presence of different salt concentrations ranging from 10% to 35%, but the optimum growth was observed at a concentration of 15% NaCl (the higher the salt concentration, the lower the growth). These results indicate that the two isolates are moderate haloarchaea compared to extreme haloarchaea such as *Haloferax mediterranei* or *Haloferax volcanii,* both requiring salt concentrations above 20% for optimum growth [5]. Apart from the C/N ratio and phosphate limitation, the appropriate concentration of NaCl has been shown to be critical to producing PHA by haloarchaea [26]. Isolated strains were also subjected to a wide pH range from acidic to alkaline conditions to examine their effect on growth and PHA production. The optimal growth of strain NRS_35 was identified at a pH value of 9, whereas strain NRS_38 grew better at a pH value of 8. Cell growth and concentrations of the total PHA produced were both reduced at the acidic pH as expected for haloarchaea (most of them thrive best at neutral or slightly alkaline pHs) [27,28,29].

Once the parameters for optimal growth were identified, the quantification and characterization of the biopolymers produced were carried out. The synthesis of PHAs by haloarchaea has been less explored than in bacterial counterparts or plants. In summary, several enzymes mediated PHA synthesis and granule formation: PhaA, PhaB, and PhaC [30]. PHA biosynthetic pathways are also linked to different pathways such as glycolysis, the Krebs Cycle, the Calvin Cycle, and β-oxidation [31]. Under nutrient-rich environments, the production of high amounts of coenzyme A from the Krebs Cycle blocks PHA synthesis by inhibiting 3-ketothiolase (PhaA) and shifts into the Krebs Cycle for energy production [31]. On the other hand, other studies analyzing the haloarchaeal genetics stated that pathways related to PHA synthesis are mainly present in those halophilic archaea with larger proteome sizes and higher GC contents [32]. More studies are still required to understand PHA biosynthesis in haloarchaea as well as the molecular mechanism involved in its regulation. During the last decade, most of the studies on PHAs in haloarchaea were focused on their production at the laboratory scale and the isolation, quantification, and characterization of biopolymers. Thus, Pramanik et al. [33] reported the production of polyhydroxybutyrate (PHB) by *Haloarcula marismortui* using vinasse as a carbon source, and Karray and coworkers [7] reported the production of PHB in *Haloarcula tradensis* using starch as a carbon source. Other *Haloarcula* species, such as *Haloarcula japonica, Haloarcula amylolytica*, and *Haloarcula argentinensis*, can also accumulate PHB [12,18]. With regards to previous studies of PHA production by *Haloarcula* sp, only two studies reported successful PHA production using starch as carbon; *Haloarcula* sp. IRU1 could produce 57% PHB/CDW [30] while Karray and coworkers [7] reported PHA accumulation of approximately 1.42% PHB/CDW by *Haloarcula* strain CEJ48-10. Other studies [13,19] described the PHA production by *Haloarcula japonica*, *Haloarcula amylolytica*, and *Haloarcula argentinensis* with yields obtained from glucose of 0.5, 4.4, and 6.5% (of CDW) [34]. *Haloarcula* sp. strain NRSA20 can accumulate PHA 23.83%, 14%, 11%, 12%, and 8% of PHB/CDW by using 10 g L^−1^ of starch, CMC, sucrose, glucose, and glycerol, respectively.

With this work, the list of haloarchaea able to produce PHA is extended, shedding light on this process and confirming that these two new isolates are highly versatile using different carbon sources for growth and PHA production. The yield of PHA production in this study is similar to those previously described from haloarchaea as well as from bacteria wildtype strains [35]. The main advantage of using haloarchaea instead of bacteria apart from cellular lysis with distilled water and avoiding sterilization is their genuine and versatile metabolism. This versatility could be mainly due to the ability to produce several hydrolytic enzymes such as α-amylases as it has been previously described from other haloarchaea. α-amylases catalyze the hydrolysis of starch and produce fructose and glucose [36,37]. These enzymes were found to be stable under saline concentrations ranging from 2 to 4 M NaCl, while at a concentration of 3 M NaCl, the enzyme exhibited the maximum activity at pH values ranging from 7 to 8 and temperatures between 50 and 60 °C [38]. Apart from glucose or starch, some haloarchaea can metabolize glycerol (an inexpensive carbon source) as a nutrient source for growth and produce PHAs [39]. In this regard, two pathways are used by haloarchaea to metabolize the glycerol: On the one hand, glycerol kinase phosphorylates glycerol to synthesize sn-glycerol-3-phosphate (G3P) and then oxidizes it using G3P dehydrogenase (G3PDH) to produce dihydroxyacetone phosphate (DHAP). On the other hand, glycerol can be oxidized using glycerol dehydrogenase to produce dihydroxyacetone (DHA), with the latter being phosphorylated using DHA kinase to DHAP [37].

*Haloferax* species are likely the better-described and most used for biotechnological purposes at the time of writing this review. The growth pattern, as well as the high productivity and quality, of PHA synthesized by the genus *Haloferax* was reported by several studies [40,41,42,43,44]. Thus, the production of PHBV by *Haloferax mediterranei*, in the presence of glucose as the only carbon source, reached up to 9%, while exceeding 20% in *Halogeometricum borinquense* E3 [45]. Furthermore, in the presence of direct precursors in the culture media such as volatile fatty acids, the 3HV molar fraction in *Haloferax mediterranei* can reach up to 99% [46]. PHBV is the most marketed biopolymer due to its physical properties, making many applications in agri-food, biomedicine, etc., possible [5]. For this reason, the characterization of more microorganisms able to produce not only PHAs, but particularly PHB or PHBV, is becoming one of the major concerns in this field of knowledge. Besides, it has also been reported that the nutritional composition of the culture media affects the composition of PHAs finally synthesized, thus making the optimization of the culture media essential to supporting a good rate of growth and significant biomass at the stationary phase of growth as well as high yields of PHV/PHBV production [5,47,48].

In order to determine the type of PHA mainly produced by these two new isolates, FTIR and LC–MS/MS analyses were carried out. The FTIR pattern (at 4000–400 cm^−1^) for both *Halolamina sediminis* sp. strain NRS_35 and the unclassified *Halolamina* sp. strain NRS_38 revealed a wide range of absorption peaks detected around 3447.71 and 3442.84 cm^−1^, thus indicating the presence of –NH or –OH groups’ bonds in protein [49,50]. The weak absorption at 2958.39 cm^−1^ in the unclassified *Halolamina* sp. strain NRS_38 (Figure 4A) is related to the C–H stretching of the –CH_3_ group in fatty acids, while the peaks around 2927.30 and 2925.87 cm^−1^ are due to symmetric stretching of the C-H bond in CH_2_ groups [51,52]. The sharp peaks identified around 1628.98 and 1629.28 cm^−1^ could be related to the carbonyl (-C=O) group on amides and related to proteins [53] or may be associated with the –CONH– group in amino sugars and proteins [50].

Moreover, the absorption around 1214.89 and 1216.14 cm^−1^ represents the availability of sulfated functional groups such as C-S-O and S-O [50,54]. The weak band around 895.30 cm^−1^ is attributed to α-D-glucose [55]. Meanwhile, the sharp bands at 764.31 and 761.73 cm^−1^ are indicative of glycosidic linkage [56]. On the other hand, the peaks at 668.45 and 671.73 cm^−1^ are related to the C-H groups of aromatic compounds [57]. The current FTIR patterns reflect the chemical composition of S-layer glycoproteins present in haloarchaea as it is composed of polysaccharides, pseudopeptidoglycan, glycoproteins, or pure protein. On the other hand, the FTIR pattern (Figure 5C) illustrates the recorded peaks for standard PHB.

Finally, the results obtained convincingly demonstrated, via LC-MS analysis, the presence of PHB synthase (PhaC), and PHB accumulation was significantly impacted by the presence of PhaC, which was detected at RT: 7.353 [58]. Consequently, it is possible to assume that the accumulation of PHB with high molecular weight is connected to the activity of PhaC as previously described in earlier research [34,59,60,61,62,63].

## 4. Materials and Methods

### 4.1. Sampling and Site Description

Sediment and brine samples were collected in September 2019 from a hypersaline environment on the southern coast of Jeddah, Saudi Arabia (21°10′16.04″ N, 39°11′5.94″ E) in 1000 mL sterile Pyrex bottles. All samples were kept at 4° C and microbiologically examined within 24 h after their collection.

### 4.2. Enrichment, Isolation, and Growth Conditions

The samples were enriched via culturing in a PHA-accumulating medium described by Han et al. (2010) [19], HSM, containing, per liter, 250 g of NaCl, 20 g of MgSO_4_.7H_2_O, 20 g of KCl, 2.0 g of trisodium citrate, 3.0 g of Na_2_CO_3_, 8.0 g of KH_2_PO_4_, 37.5 mg of FeSO_4_.7H_2_O, and 50 mg of MnCl_2_.4H_2_O. The medium was supplemented with 10 g/liter of glucose and glycerol separately, as carbon sources, buffered with MOPS, and incubated at 37 °C for 7–14 days at 150 rpm. Samples were serially diluted, and 1 mL of each dilution was plated onto HSM as described above and supplemented with agar (20 g L^−1^) to isolate halophilic archaeal strains accumulating PHA. Pure isolates were obtained and maintained through repeated culture on HSM.

### 4.3. Screening for PHA-Producing Haloarchaeal Isolates

After two weeks of incubation at 37 °C, ten different halophilic archaeal isolates were stained with Sudan Black B (Sigma) to determine their potential as PHA producers, as described by Murray and coworkers [64]. At the early stationary phase of growth, an aliquot of culture was smeared on a clean glass slide, then heat-fixed and stained with Sudan Black B (3% w/v in 70% ethanol) for 10 min, and finally, immersed in xylene until completely decolorized. The samples were rinsed and dried after being counterstained with safranin (Sigma; 5% (w/v), aqueous solution) for 10 s. Phase contrast microscopy (Nicon Eclips E600) was used to investigate the cells. Positive strains, indicating PHA producers, were recognized as blue-black cells under the microscope, with the bacterial type of strain *Escherichia coli* (ATCC35218) as a negative control.

### 4.4. Identification of the Potential Strains

Following the instructions provided by the manufacturer, a DNA extraction kit (QI-AGEN, Hilden, Germany) was used to extract genomic DNA for molecular identification of the selected isolates. The 16S rRNA gene was amplified using Archaea-universal primers (Invitrogen, USA), 5′-ATT CCG GTT GAT CCTGCC GG-3′ primers (positions 6–25), and 5′AGG AGG TGA TCC AGC CGC AG-3′ primers (positions 1540–1521), as described by Ventosa and coworkers [65]. The conditions of PCR were as follows (50 μL reaction): 30 cycles, 95 °C pre-denaturation for 5 min, 94 °C denaturation for 1 min, 60 °C annealing for 1 min, 72 °C extension for 1 min 30 s, 72 °C final extension for 10 min, 4 °C hold. A total of 50 ng/l of PCR products were delivered to MacroGen Company in Seoul, Korea, according to their specifications. The sequences were analyzed using BLAST (http://www.ncbi.nlm.nih.gov/BLAST, accessed on 1 October 2022) to obtain a preliminary identification of the strains. The cluster analysis was carried out using the MEGA X version 10.2.6.

### 4.5. Optimization of Growth Conditions of the Potential PHA-Producing Haloarchaeal Strains

A total of 100 ul of the culture at the exponential phase of growth was used as the inoculum for 100 mL of the PHA production media: Previous media supplemented with glycerol (1% (*v*/*v*) and incubated at 37 °C with an agitation rate of 150 rpm. Cellular growth was measured every 48 h for up to two weeks using a spectrophotometer set to 600 nm. The effects of different conditions of glycerol on PHA production by the potential strains were examined. The effect of other parameters such as pH (5, 6, 7, 8, and 9), temperature (4, 20, 37, 45, 55, and 65 °C), and NaCl concentration (100, 150, 200, 250, 300, and 350 g L^−1^) were also explored. In addition, the effect of different carbon sources such as starch, carboxymethyl cellulose (CMC), sucrose, glucose, and glycerol on PHA production by the selected strains was examined under optimal growth conditions. Each carbon source was filtered separately and then added to the sterilized production medium at a final concentration of 10 g L^−1^. All the conditions were tested in triplicate.

### 4.6. Extraction of the Polymer

The cultures previously described were grown until the stationary phase and later centrifuged for 25 min at 5000 rpm to harvest the cells. The dry weight of the pellets was obtained, and the pellets were then treated as follows. To recover PHAs granules, the pellets were resuspended in an equal amount of 6% (w/v) sodium hypochlorite and incubated at 37° C for 10 min. After this incubation, the mixture was centrifuged at 5000 rpm for 30 min. Before being treated with hot chloroform, the pellets containing the granules were washed in acetone and ethanol (30:70). After the pellets were dissolved in chloroform, Whatman filter paper (grade 1, Cat No 1001-110) was used to filter out the cell debris, thus making the presence of PHA in the chloroform solution possible. Finally, the filtrate was evaporated in a hot air oven at 40 °C, and the dry weight of the extracted polymer was determined [66]. The percentage of PHAs accumulation was calculated using the following formula, according to Sathiyanarayanan and Munir [67,68]:

Dry weight of extracted PHB/PHA (g/mL) × 100/Cell Dry weight of biomass (CDW)

Cell dry weight (CDW) = weight of falcon tube with dried pellets—the weight of empty falcon tube.

Dry weight of extracted PHA = weight of filter paper with dried filtered PHA—weight of empty filter paper.

### 4.7. Characterization of the Biopolymer by FTIR

The functional groups CH, CH_2_, CH_3_, C=O, C-O, and OH, which are significant determinants for the existence of PHAs in the extracted biopolymer, were subjectively identified and detected using FTIR spectroscopy (Perkin Elmer Spectrum GX Range Spectrometer, Bridgeport Avenue, Madison, USA), according to the method described by Mohapatra et al. [69].

### 4.8. Characterization of Biopolymer by LC–MS/MS

An extracted biopolymer from the NRS35 and NRS38 strains was identified using the LC-MS/MS systems of AB Sciex (Spectralab Scientific Inc., Markham, North America) linked to an API 5000 Triple Quadrupole mass spectrometer. The isocratic elution was carried out, with a flow rate of 0.003 mL min^−1^ of the mobile phase (0.001 M chloroform). A UV detector (10 AC) operating at 210 nm identified the presence of PHB. The sample was injected at a rate of 5.0 μL/s and detected over the course of 11.5 min. Using commercial PHB as the control (Sigma-Aldrich, St. Louis, MO, USA), the data were analyzed using the Mass Hunter software. The analytical column used was the Thermo Scientific™ Hypersil™ ODS (C18) Column, with a 10 μm particle size, and the temperature was maintained using a column heater [70,71].

### 4.9. Statistical Analysis

Three replicates were conducted for each analysis (n = 3). The measurements were displayed as the mean ± standard deviation (SD). IBM^®^ SPSS was used to conduct the statistical analysis utilizing an analysis of variance (ANOVA) on normally distributed datasets with a 95% confidence interval (IBM Corp. in Armonk, NY, USA. Chicago, IL, USA). *p* < 0.05 was considered to indicate a significant difference. The Duncan post hoc test was used to compare the significant difference between the means of the tested factor.

## 5. Conclusions

The promising haloarchaeal strains, *Halolamina sediminis* strain NRS_35 (OL912954) and *Halolamina* sp. strain NRS_38 (OL912955), were obtained in pure culture from the Solar Saltern on the southern coast of Jeddah, Saudi Arabia. Both strains were able to use starch, CMC, sucrose, glucose, and glycerol as the sole carbon sources for PHB production. The highest yield of PHB synthesis by strains NRS_35 and NRS_38 was 33.4% and 27.29%, respectively, using starch as a carbon source at 37 °C, pH 7, and 25% NaCl (w/v). This yield is similar to the average reported from other haloarchaea and bacteria, and it could be improved in the future by optimizing nutritional conditions or by designing mutants able to overproduce PHB. FTIR pattern and LC-MS analyses revealed significant groups that are typical characteristics of PHB. Future research will focus on optimization employing other low-cost feedstocks to improve both quality and PHB productivity.

## Figures and Tables

**Table 1:** Phenotypic characteristics of the studied haloarchaeal strains.

Characteristics	Strains	
	NRS_35	NRS_38
Pigmentation	Red	Orange-red
Cell morphology	Pleomorphic	Pleomorphic
Motility	+	-
NaCl range (%)	10–35	10–35
Temp. range (°C)	30–65	25–45
pH range	6.0–9.0	7.0–9.0
H_2_S production	-	-
**Hydrolysis of:**		
Gelatin	-	-
Casein	-	+
Starch	+	+
Tween 80	-	-
**Carbon sources for growth:**		
D-Galactose	-	+
DL-Lactate	+	-
D-Mannose	+	+
Pyruvate	-	+
Acetate	+	+
Glycine	+	-
DNA G+C content (mol%)	59.0	57.0

+, detectable; -, not detectable.

**Table 2:** Analysis of variance (ANOVA) of using different carbon sources on PHA production.

Carbon Source	Strain NRS_35 (mg/L)	Strain NRS_38 (mg/L)
Starch	41.67 ± 2.10 ^a^	36.63 ± 3.20 ^a^
CMC	11.25 ± 1.08 ^b^	14.789 ± 1.55 ^b^
Sucrose	10.65 ± 1.03 ^b^	10.647 ± 1.40 ^c^
Glucose	8.99 ± 0.98 ^b^	12.587 ± 2.14 ^b^
Glycerol	14.33 ± 1.42 ^c^	11.458 ± 1.08 ^b^

Data represent mean ± SD from three replicates. In each column, ^a,b,c^ different superscript letters indicate significant differences at the *p* < 0.05 level (one-way ANOVA and Duncan post hoc test).

## Data Availability

Not applicable.
